# Substantial variability and inconsistent quality of publicly available rehabilitation protocols after quadriceps tendon anterior cruciate ligament reconstruction: A cross‐sectional analysis of academic orthopaedic surgery programmes

**DOI:** 10.1002/jeo2.70653

**Published:** 2026-02-13

**Authors:** David Slawaska‐Eng, Caitlin Svendsen, Emily Zhang, Kanika Tibriwal, Dan Cohen, Lauren Gyemi, Sachin Tapasvi, Matthieu Ollivier, Darren de Sa

**Affiliations:** ^1^ Division of Orthopaedic Surgery, Keck School of Medicine University Southern California Los Angeles California USA; ^2^ Division of Orthopaedic Surgery, Department of Surgery McMaster University Hamilton Ontario Canada; ^3^ Division of Otolaryngology University of Alberta Edmonton Alberta Canada; ^4^ Faculty of Medicine University of British Colombia Vancouver British Colombia Canada; ^5^ Division of Orthopaedic Surgery University of Pittsburgh Medical Center (UPMC) Pittsburgh Pennsylvania USA; ^6^ The Orthopaedic Speciality Clinic Pune India; ^7^ Department of Orthopaedic Surgery, Institute for Locomotion Aix‐Marseille University Marseille France; ^8^ Department of Biomechanics, APHM, CNRS, ISM, St Marguerite Hospital, Institute for Locomotion Aix‐Marseille University Marseille France

**Keywords:** anterior cruciate ligament reconstruction, physical therapy modalities, quadriceps tendon, rehabilitation, return to sport

## Abstract

**Purpose:**

Quadriceps tendon (QT) autograft is increasingly used for anterior cruciate ligament reconstruction (ACLR), yet rehabilitation guidelines remain extrapolated from patellar tendon (PT) or hamstring tendon (HT) protocols. This cross‑sectional study evaluated publicly available postoperative rehabilitation protocols from academic orthopaedic programmes to describe their content, assess variability and identify key trends.

**Methods:**

Accredited orthopaedic residency programmes were identified through the Electronic Residency Application Service (ERAS) and Canadian Resident Matching Service (CaRMS). A three‐step systematic web‐based search was conducted to identify publicly available QT‐ACLR rehabilitation protocols. Protocols were included if specific to QT autografts and excluded if addressing concomitant meniscal repairs. Two independent reviewers extracted data on rehabilitation components and timelines. The proportion of protocols including each component and the median initiation time were calculated.

**Results:**

Of 219 programmes screened, 16 eligible protocols were identified. Nine were QT‐specific and seven were general ACLR protocols. Key trends included: (1) use of a hinged brace locked in extension for 2–4 weeks (86.7%); (2) initiation of icing, cryotherapy and patellar mobilizations immediately postoperatively (68.8%); (3) neuromuscular electrical stimulation use within the first 4 weeks (56.2%); (4) target of full extension by 2–4 weeks and flexion by 3–4 months (100%); (5) strengthening, balance and proprioceptive training beginning between 1–3 months (93.8%–100%) and (6) return‑to‑sport (RTS) testing between 5 and 9 months, using time‐based and/or criterion‐based recommendations (100%). Substantial variability existed in exercise prescriptions, adjunctive therapy use and RTS criteria.

**Conclusions:**

Publicly available QT‐ACLR rehabilitation protocols from academic programmes emphasize early weight‐bearing, range of motion restoration and progressive strengthening but reveal considerable variability in timing, adjunctive therapies and RTS guidelines. Many protocols mirror those for PT and HT autografts rather than being tailored to QT‐specific considerations. Standardized, evidence‐based rehabilitation guidelines that address graft‐specific risks, psychological readiness and telehealth delivery are needed to optimize outcomes following QT‐ACLR.

**Level of Evidence:**

Level IV.

**Abbreviations**
ACL: anterior cruciate ligament
ACLR: anterior cruciate ligament reconstruction
BFR: blood flow restriction
CaRMS: Canadian Resident Matching Service
ERAS: Electronic Residency Application Service
HT: hamstring tendon
NMES: neuromuscular electrical stimulation
PT: patellar tendon
QT: quadriceps tendon
ROM: range of motion
RTS: return to sport
SQuASH: soft‐tissue quadriceps autograft ACL‐reconstruction in the skeletally immature versus hamstrings (trial)

## INTRODUCTION

In recent years, the use of quadriceps tendon (QT) autograft for anterior cruciate ligament reconstruction (ACLR) has gained considerable attention, bolstered by emerging evidence supporting it as an efficacious and versatile fixation method as a full‐ or partial‐thickness graft, and with or without bone plug for both primary and revision ACLR procedures [4, 5, 12, 18, 20]. The promising biomechanical properties and clinical outcomes associated with QT‐ACLR highlight its potential benefits [3]. The distinctive factors associated with QT‐ACLR, including decisions related to partial‐ versus full‐thickness graft harvest, graft width and the use of a patellar bone block, introduce unique considerations for both surgical and rehabilitative phases.

QT autograft has emerged as a versatile option for ACLR, offering comparable biomechanical strength and clinical outcomes to traditional patellar tendon (PT) and hamstring tendon (HT) grafts [5, 18, 20]. The graft may be harvested as a partial‑ or full‑thickness tendon with or without a bone block, and its appeal is enhanced by a low incidence of donor‑site morbidity [12]. Despite these advantages, the unique surgical considerations of QT‑ACLR, such as harvest thickness, graft diameter and fixation technique, introduce specific rehabilitation challenges, including potential extensor mechanism weakness and quadriceps inhibition.

The unique surgical nuances of QT‐ACLR have not been well studied, particularly when optimizing postoperative rehabilitation and return to sport (RTS). While numerous reviews and evidence‐based guidelines exist for postoperative rehabilitation following ACLR with PT and HT grafts [8, 10, 15, 23], there is a relative paucity of literature addressing the specifics of postoperative rehabilitation for QT‐ACLR. There remains a notable gap in high‐quality evidence surrounding QT‐ACLR rehabilitation, particularly whether using postoperative protocols similar to other graft options will lead to comparable and optimal outcomes [3, 19, 26]. Concerns over the potential for extensor mechanism disruption and its impact on quadriceps weakness and range of motion (ROM) are ongoing topics of discussion, despite recent literature supporting rates and types of postoperative complications comparable to other graft choices [19]. Furthermore, existing evidence regarding which elements and activities contribute to optimal outcomes and a safe RTS for QT‐ACLR rehabilitation is also limited. This further emphasizes the need for standardized and effective rehabilitation protocols for QT‐ACLR.

The objective of this study was to perform a cross‑sectional evaluation of publicly available postoperative rehabilitation protocols for QT‐ACLR to identify current trends and highlight areas where further high‐quality research is needed. It was hypothesized that, analogous to protocols for PT and HT autografts, the majority of QT‐ACLR rehabilitation protocols would prioritize achieving full ROM (particularly extension), early weight‐bearing and progression through closed and open chain exercises, with a focus on RTS in the later phases of rehabilitation.

## METHODS

A cross‑sectional descriptive analysis of postoperative rehabilitation protocols for QT‐ACLR was performed from accredited academic orthopaedic surgery programmes in the United States of America and Canada. Programmes listed on the Electronic Residency Application Service (ERAS) and the Canadian Resident Matching Service (CaRMS) were identified and an extensive search for publicly available rehabilitation protocols was conducted. Institutional review board approval and formal registration were not required because the study analysed publicly available documents and did not involve human participants.

Consistent with prior work evaluating online rehabilitation protocols [13, 14], a three‑step web‑based search was performed. First, the ERAS and CaRMS programme websites were searched using the terms ‘ACL rehabilitation protocol’, ‘quadriceps tendon protocol’ and ‘QT ACLR protocol’. Second, a Google search was conducted combining each programme's name with the keywords ‘quadriceps tendon ACL rehabilitation protocol’. Third, protocols authored by surgeons affiliated with each programme were searched for, using combinations of the surgeon's name and ‘ACL rehabilitation protocol’ with ‘quadriceps tendon’ or ‘QT’. The search was restricted to protocols written in English and publicly available without password protection.

Protocols were included if they met the following criteria: they were publicly available, were specific to QT autografts for ACLR and did not involve concomitant meniscal tear repair. Inclusion of protocols was restricted from ERAS/CaRMS‐accredited academic programmes or their affiliated surgeons to (i) sample protocols most likely to be peer‐reviewed internally and broadly disseminated to trainees and (ii) reduce heterogeneity from private‐clinic marketing materials or third‐party sites. Protocols were excluded if they did not meet these criteria or were from non‐ERAS or non‐CaRMS programmes.

Two independent reviewers examined all protocols that met the inclusion criteria. Discrepancies between reviewers were resolved through discussion with a senior author. Rehabilitation components from each protocol were categorized as outlined in Table 1. The primary outcome was the presence of these rehabilitation components in the protocols, while the secondary outcome was the recommended timing for each component. The proportion of protocols that included each component was determined, and the median postoperative week for the initiation of each component across all included protocols was calculated.

**Table 1:** Rehabilitation protocol components.

Protocol component	General recommendations
Prehabilitation	Effusion reduction, range of motion, strengthening and straight leg raises
Post operative adjunctive therapy	Bracing, patellar mobilization, neuromuscular electrical stimulation, ice/cryotherapy, heat therapy, ultrasound and blood flow restriction
Early motion and weight‐bearing	Flexion/extension goals, weight‐bearing parameters, hamstring stretch, quadriceps stretch, calf stretch, heel/wall slides, prone hangs, posterior tibial mobilizations and stationary bike
Strengthening	Straight leg raise, quadricep sets/isometrics, hamstring curls, step‐up/down, leg press, heel slides, lunges, closed‐chain knee exercises, single‐leg squat, ‘mini’ squat, full squat, hip/core exercises, calf raises, toe raises, deadlift and open‐chain knee exercises
Proprioception	Weight shifting, straight leg balance, balance board, marching/step and hold and perturbations
Functional testing	Single‐hop, three‐hop, vertical hop, horizontal hop, triple crossover, Y‐balance, isometric quadriceps strength, isometric hamstring strength and one‐repetition max test
Return to activity/sport	Treadmill walking, jogging, running, sprinting, stationary bike, elliptical, stair climber, swimming, plyometric/sport‐specific training, cutting/pivoting drills, jumping and agility

## RESULTS

**Figure 1:**
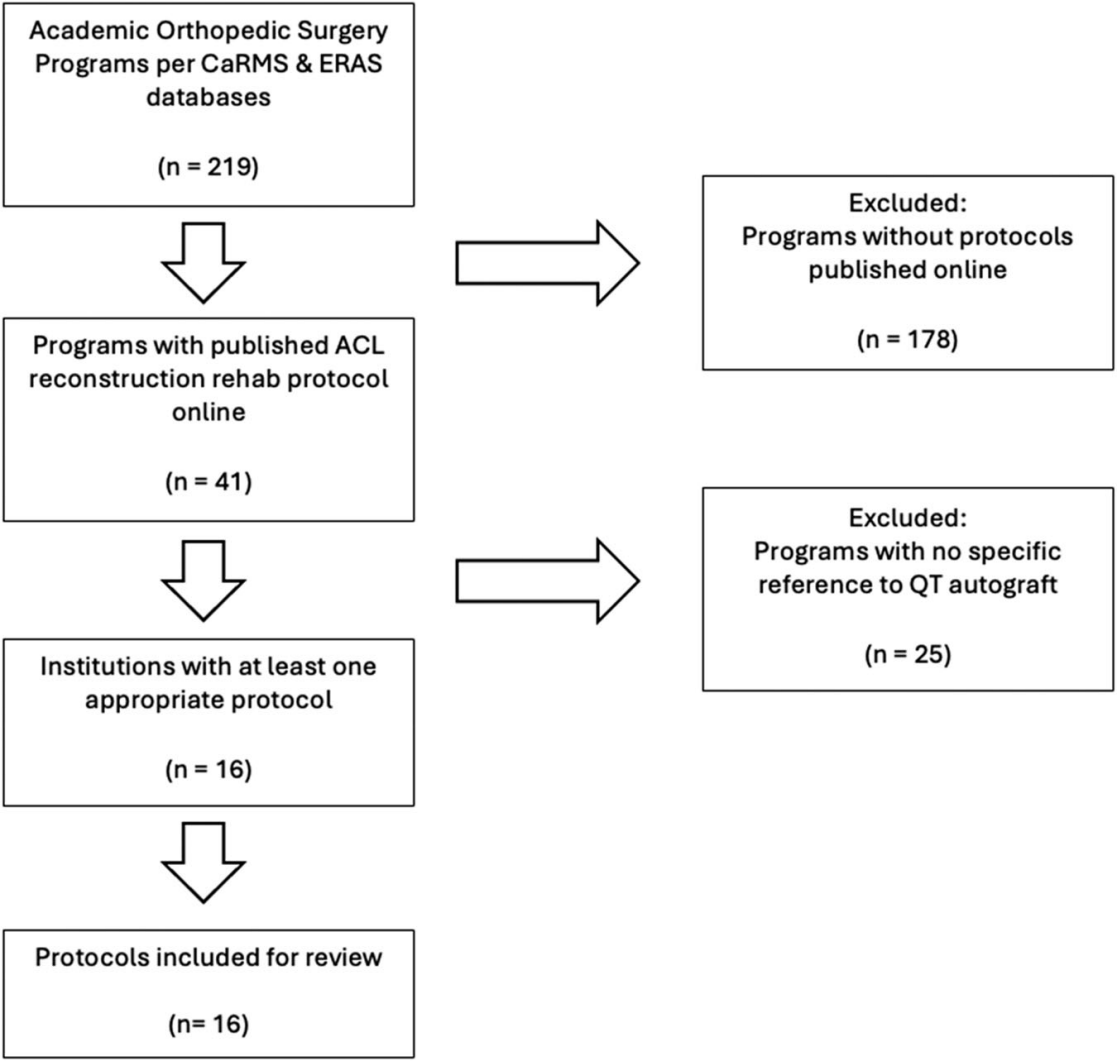
Flowchart demonstrating search process. ACL, anterior cruciate ligament; CaRMS, Canadian Resident Matching Service; ERAS, Electronic Residency Application Service; QT, quadriceps tendon.

A total of 219 programmes (202 ERAS and 17 CaRMS) were identified within the ERAS and CaRMS databases, and 41 of these programmes were found to have available ACLR protocols. Of these programmes, 25 were excluded due to no specific reference to QT autograft. Ultimately, 16 protocols were assessed (Figure 1). Of the 16 selected protocols, 14 utilized time‐based recommendations, while 2 utilized phase‐based recommendations. Nine protocols provided rehab protocols specific to QT‐ACLR, while seven provided protocols not specific to using the QT.

### Graft configuration reporting

None of the 16 protocols stratified rehabilitation recommendations by quadriceps graft configuration (full‐ vs. partial‐thickness harvest) or by the presence/absence of a patellar bone block. No protocol offered configuration‐specific restrictions or timelines.

### Prehabilitation

Three (18.8%) of the 16 protocols included preoperative instructions. These protocols emphasized joint oedema/effusion reduction, ROM exercises and lower extremity strengthening, particularly the ability to perform a straight leg raise. All three protocols highlighted the importance of achieving full knee ROM and the ability to perform straight leg raises preoperatively.

### Postoperative adjunctive therapy

**Figure 2:**
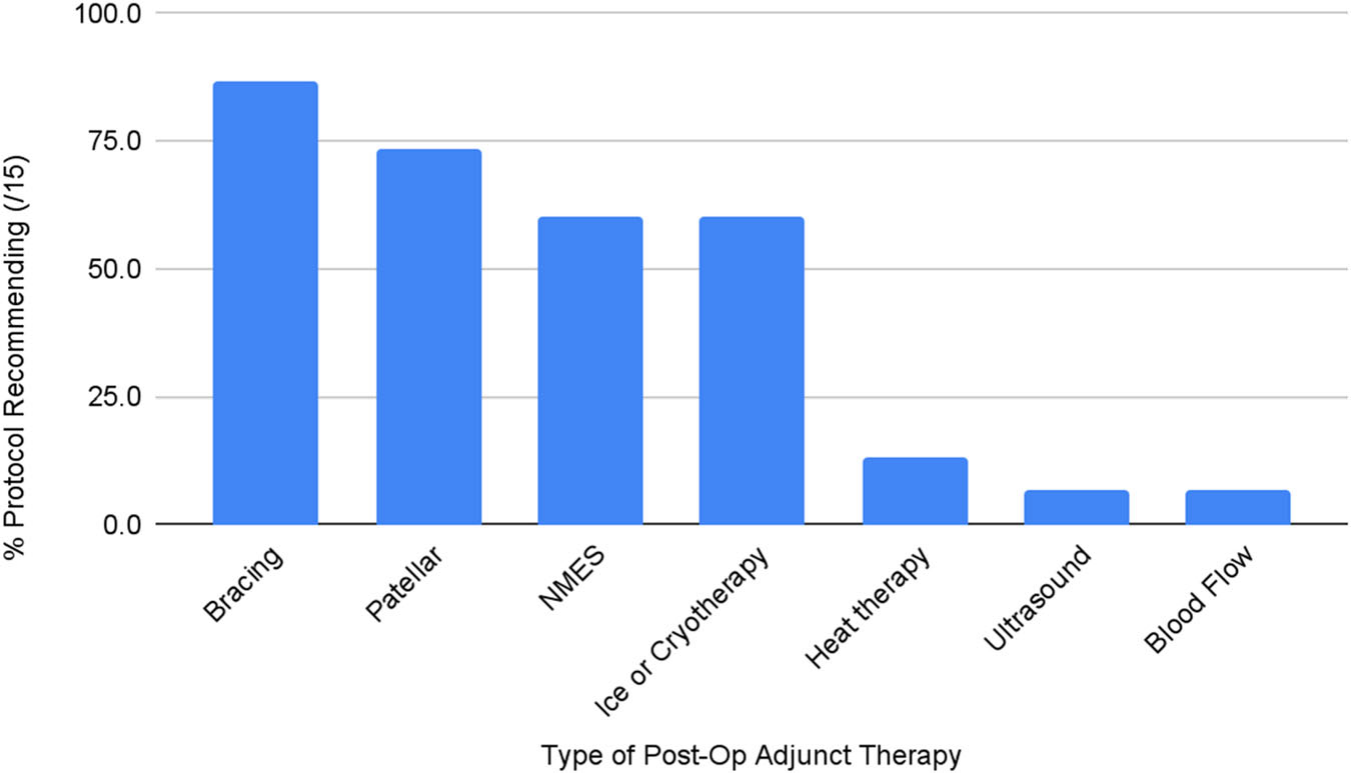
Types of postoperative adjunctive therapies. NMES, neuromuscular electrical stimulation.

Moreover, 15 of the 16 protocols included in this study discussed postoperative adjunctive therapies, with a total of six therapies being identified either in combination or as monotherapy (Table 2; Figure 2). Specific therapies included knee bracing, icing/cryotherapy, heat therapy, neuromuscular electrical stimulation (NMES), ultrasound and blood flow restriction (BFR). Bracing was the most recommended therapy (86.7%), followed by patellar mobilizations (73.3%), NMES (60.0%) and icing/cryotherapy (60.0%). Most protocols recommended combining multiple therapies, with 33% recommending bracing, NMES and icing/cryotherapy. Dual therapy combinations included bracing with icing/cryotherapy (20.0%) or NMES (20.0%). Monotherapy recommendations were less common (bracing alone [13.3%], NMES alone [6.7%] or icing/cryotherapy alone [6.7%]).

**Table 2:** Types of postoperative adjunctive therapies.

Type of postoperative adjunct therapy	% Protocol recommending (/15)
Bracing	86.7
Patellar mobilization	73.3
NMES	60.0
Ice or cryotherapy	60.0
Heat therapy	13.3
Ultrasound	6.7
Blood flow restriction	6.7

Abbreviation: NMES, neuromuscular electrical stimulation.

#### Bracing

Thirteen protocols (86.7%) recommended postoperative bracing. Ten protocols recommended the use of a hinge brace, while two protocols recommended a custom ACL brace. Among these, six (46.2%) recommended wearing the brace in full extension for ambulation up to 1–2 weeks post‐surgery, while four protocols (30.8%) suggested up to 4 weeks. One protocol (7.7%) recommended bracing in extension for up to 6 weeks. For sleeping, four protocols (30.8%) each recommended bracing in full extension for either 1 week (50%) or 4 weeks (50%). Discontinuation of bracing varied: 7.7% suggested within the first 2 weeks, 15.4% within the first 4 weeks and 23.1% recommended 4–6 weeks. Some protocols included other criteria for discontinuing the brace, such as achieving a ROM of 0°–90°, tolerating full weight‐bearing, normal gait or performing a straight leg raise.

#### NMES

Nine protocols (56.2%) recommended NMES. Of these, six (66.7%) provided specific time‐based guidelines, all within the first 4 weeks postoperatively. One protocol instructed patients to use NMES at 60° flexion. Detailed guidelines for NMES use were generally lacking.

#### Icing and cryotherapy

Nine protocols (56.2%) recommended icing and cryotherapy, with initiation generally starting immediately postoperatively and continuing throughout rehabilitation. Most protocols advised using this therapy for 20–30 min each hour postoperatively, then reducing to 2–3 times daily, including after physiotherapy sessions. Two protocols specifically recommended the use of a continuous cooling device in the first week after surgery, and two protocols recommended the use of the Cryo‐cuff device.

#### Patellar mobilizations

Eleven protocols (68.8%) recommended initiating patellar mobilizations postoperatively within the first 4 weeks, and six (54.5%) recommended starting within the first 2 weeks.

#### BFR

One protocol recommended BFR but did not specify any actionable BFR parameters.

### ROM

**Figure 3:**
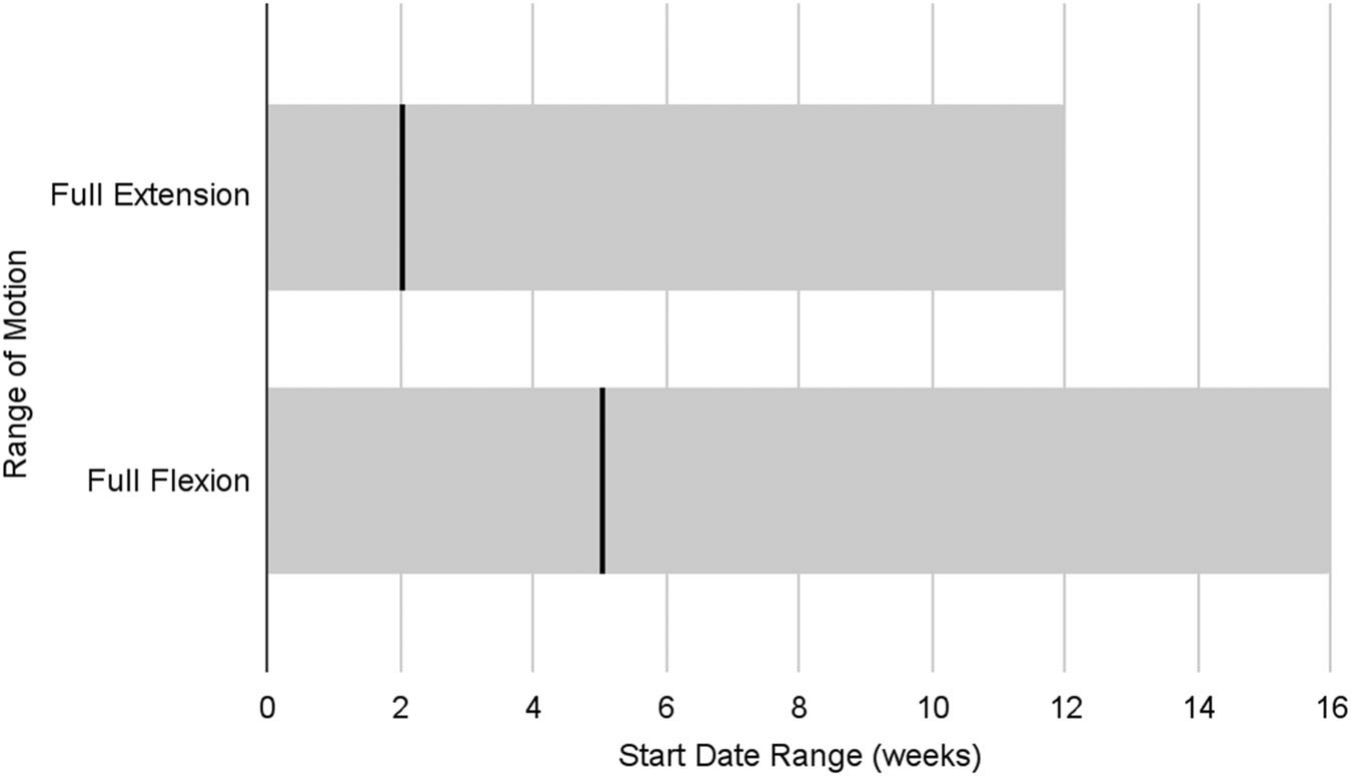
Range of motion timeline. Black lines indicate medians, with ranges shown in grey.

All 16 protocols included guidelines for postoperative knee extension and/or flexion. Of the 15 protocols that included recommendations for achieving full extension, seven (46.7%) recommended achieving full extension by 2 weeks, four (26.7%) by 4 weeks, three (20%) by 4–8 weeks postoperatively and one (6.7%) by 8–12 weeks postoperatively. Thirteen protocols included recommendations for knee flexion and unanimously suggested achieving full flexion within 16 weeks, with varying timelines. Specifically, four protocols (30.8%) were recommended within 4 weeks, five (38.5%) within 6–8 weeks, three (23.1%) within 8–12 weeks and one protocol was recommended within 4–16 weeks (Table 3; Figure 3). One protocol did not provide specific time‐based recommendations for ROM. Thirteen protocols recommended specific stretches or exercises to improve ROM, such as heel slides, seated knee active, ROM, prone hangs, wall slides, posterior tibial mobilizations and stationary bikes. One protocol recommended using a continuous passive ROM device for 6 h a day starting the first day after surgery and increased 10°–15° or as tolerated for 1–2 weeks following surgery.

**Table 3:** ROM recommendations.

ROM	Start date range (weeks)	Individual ranges	Median (weeks)
Full extension	0–12	[0–1]; [0–2]; [0–2]; [0–2]; [0–2]; [0–2]; [0–2]; [0–4]; [0–4]; [0–4]; [1–4]; [4–8]; [4–8]; [8–12]	2
Full flexion	0–16	[0–4]; [0–4]; [0–6]; [2–4]; [2–4]; [3–6]; [4–6]; [4–6; [6–8]; [8–12]; [8–12]; [10–12]; [4–16]	5

Abbreviation: ROM, range of motion.

### Weight‐bearing

All 16 protocols provided recommendations for postoperative weight‐bearing status. Fourteen protocols recommended crutches as a gait aid. In addition, 13 of the 16 protocols (81.3%) allowed weight‐bearing as tolerated immediately post‐surgery with crutches and/or a brace. Eleven of the fourteen protocols specified that patients must wear a brace locked in full extension while ambulating for varied lengths of time, ranging from 1 to 6 weeks. One protocol suggested partial weight‐bearing for the first 2 weeks, another for 1–4 weeks and the third did not provide a time‐based recommendation. Recommendations for achieving full weight‐bearing and normal gait pattern varied among the 16 protocols: 37.5% within the first 2 weeks, 37.5% within the first 4 weeks and 6.3% from Weeks 4–12. Two protocols did not provide time‐based recommendations.

### Strength training

**Figure 4:**
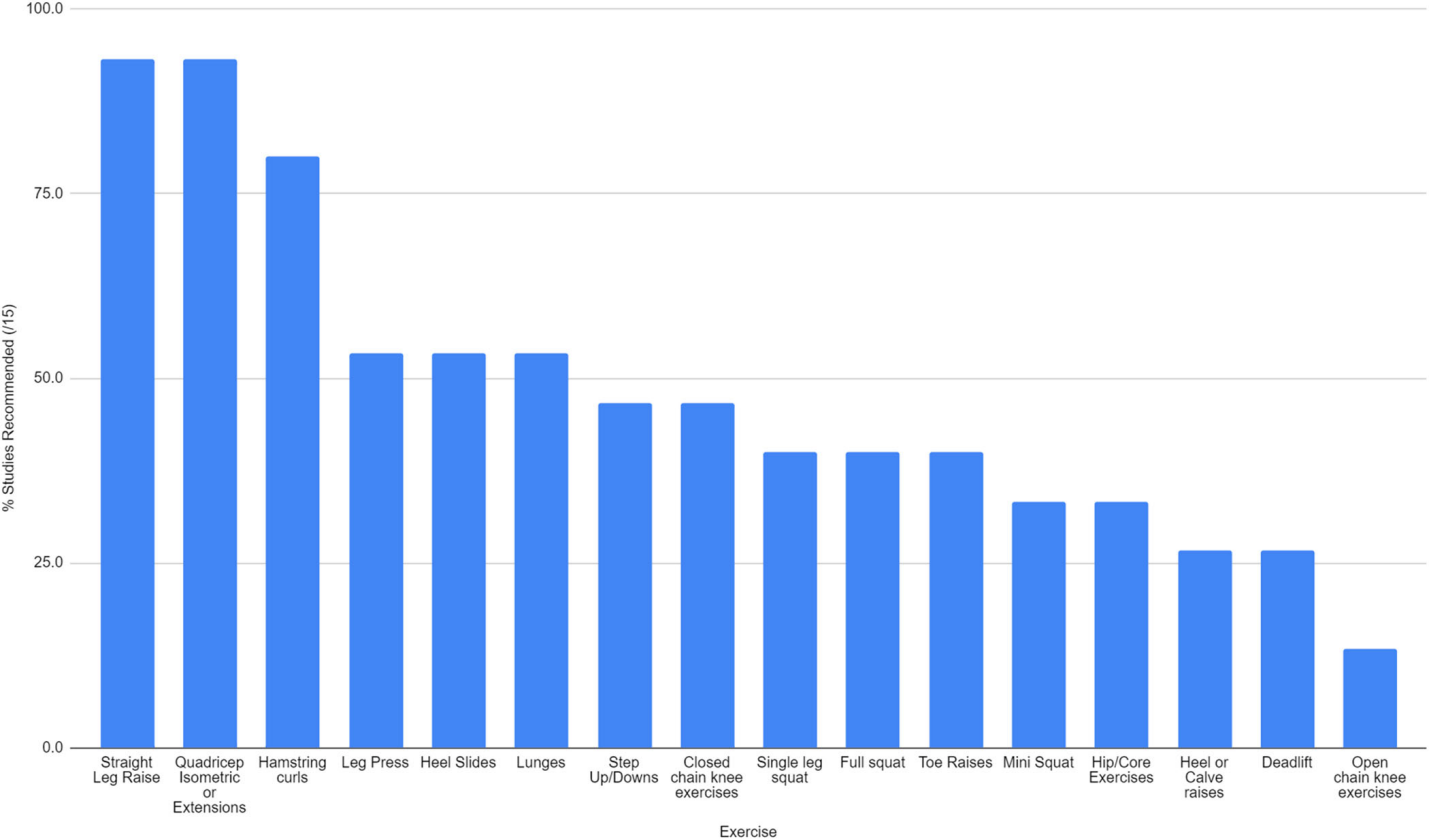
Strengthening exercise recommendations.

**Figure 5:**
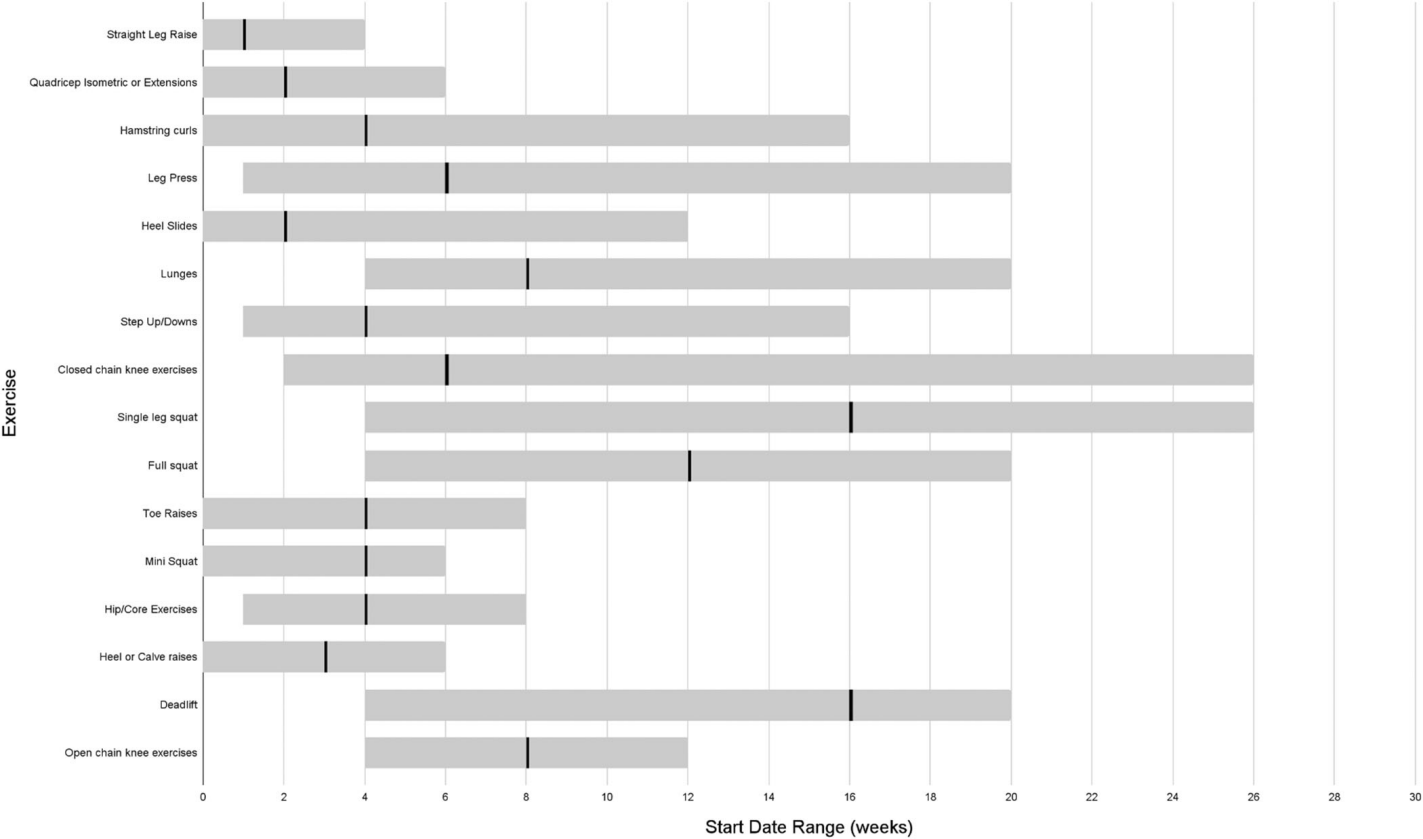
Strengthening exercise timeline. Black lines indicate medians, with ranges shown in grey.

Fifteen protocols (93.8%) included strength training recommendations, identifying 16 exercises recommended by at least two protocols (Table 4; Figure 4). Each protocol included an average of 7.6 exercises (range: 4–13). Most guidelines lacked specific instructions on exercise performance, with only four protocols providing details on repetitions, sets or resistance. Recommendations and timing varied significantly, and only two protocols offered clear instructions on exercise execution (Figure 5). Only one protocol differentiated between concentric versus eccentric knee exercises, specifically recommending concentric‐focused knee extensions. Only 2 of the 15 protocols explicitly mentioned open‐chain exercises, whereas seven protocols mentioned closed‐chain exercises. Closed‐chain movements (e.g., leg press, step‐ups) were introduced earlier, typically within 2–6 weeks (median 6), whereas open‐chain knee extensions appeared later, around 4–12 weeks (median 8).

**Table 4:** Strengthening exercise recommendations.

Exercise	% Studies recommended (/15)	Start date range (weeks)	Individual ranges	Median (weeks)
Straight leg raise	93.3	0–4	[0–1]; [1]; [0–2]; [0–2]; [0–2]; [0–2]; [0–4]; [0–4]; [0–4]; [0–4]; [0–4]; [0–4]; [1–2]; [1–4]	1
Quadricep isometric or extensions	93.3	0–6	[0–2]; [0–2]; [0–2]; [0–4]; [0–4]; [0–4]; [0–4]; [0–4]; [0–4]; [1–4]; [1–4]; [1–4]; [2–4]; [2–6]	2
Hamstring curls	80.0	0–16	[0–4]; [0–4]; [0–4]; [0–4]; [0–4]; [1–4]; [1–4]; [3–6]; [4–6]; [4–8]; [4–8]; [4–16]	4
Leg press	53.3	1–20	[1–4]; [2–4]; [2–4]; [2–6]; [3–6]; [6–12]; [6–16]; [16–20]	6
Heel slides	53.3	0–12	[0–2]; [0–4]; [0–4]; [0–4]; [1–4]; [1–4]; [1–4]; [4–12]	2
Lunges	53.3	4–20	[4–8]; [4–16]; [4–16]; [6–12]; [6–16]; [7–12]; [16–20]	8
Step up/downs	46.7	1–16	[1–4]; [2]; [2–6]; [2–6]; [3–6]; [4–8]; [4–16]	4
Closed‐chain knee exercises	46.7	2–26	[2–6]; [4–6]; [4–8];[4–8]; [6–26]; [9–12]	6
Single leg squat	40.0	4–26	[4–16]; [8–12]; [12–16]; [16–20]; [13–26]; [13–26]	16
Full squat	40.0	4–20	[4–8]; [7–12]; [4–16]; [6–16]; [16–20]	12
Toe raises	40.0	0–8	[0–4]; [0–4]; [4–6]; [4–8]; [4–8]	4
Mini squat	33.3	0–6	[0–4]; [1–4]; [2–6]; [2–6]; [3–6]	4
Hip/core exercises	33.3	1–8	[1–2]; [1–4]; [2–6]; [3–6]; [4–8]	4
Heel or calf raises	26.7	0–6	[0–2]; [2–4]; [3–6]	3
Deadlift	26.7	4–20	[4–16]; [12–16]; [16–20]	16
Open chain knee exercises	13.3	4–12	[4–8]; [12]	8

### Balance and proprioception

**Figure 6:**
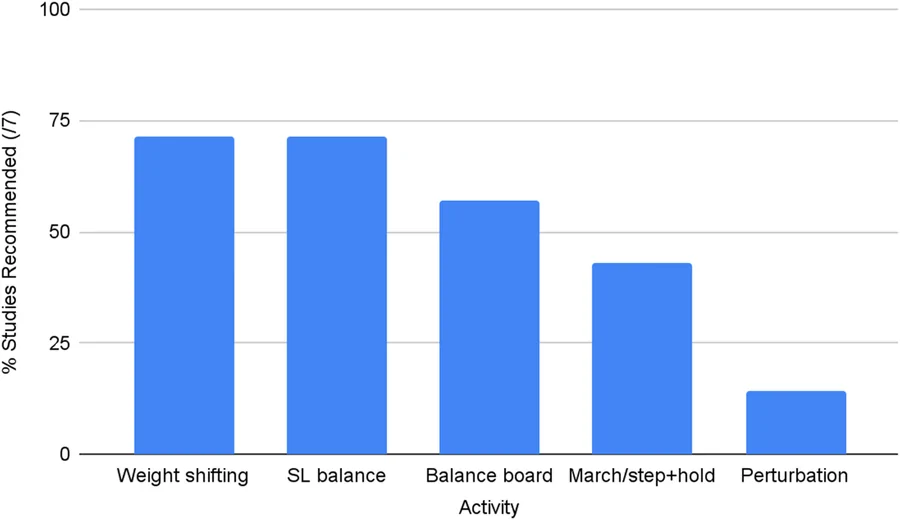
Proprioception and balance exercise recommendations. SL, single leg.

All 16 protocols included balance and proprioceptive training guidelines, with varying details (Table 5; Figure 6). Nine protocols (56.2%) provided generalized time‐based recommendations for starting this training, with seven providing specific exercises. A total of five proprioception exercises were suggested among these seven protocols, with five protocols (71.4%) recommending weight shifting, five (71.4%) single‐leg balance, four (57.1%) balance board, three (42.9%) march/step and hold and one protocol (14.3%) recommending perturbations.

**Table 5:** Proprioception and balance exercise recommendations.

Activity	% Studies recommended (/7)	Start date range (weeks)	Individual ranges	Median (weeks)
Weight shifting	71.4	0–6	[0–4]; [0–4]; [2–6]; [2–6]; [2–6]	4
SL balance	71.4	0–20	[0–4]; [4–8]; [12–16]; [16–20]	12
Balance board	57.1	0–12	[0–2]; [4–12]; [8–12]	8
March/step + hold	42.9	4–20	[4–12]; [8–12]; [16–20]; [16–20]	16
Perturbation	14.3	7–12	[7–12]	9.5

Abbreviation: SL, single leg.

**Figure 7:**
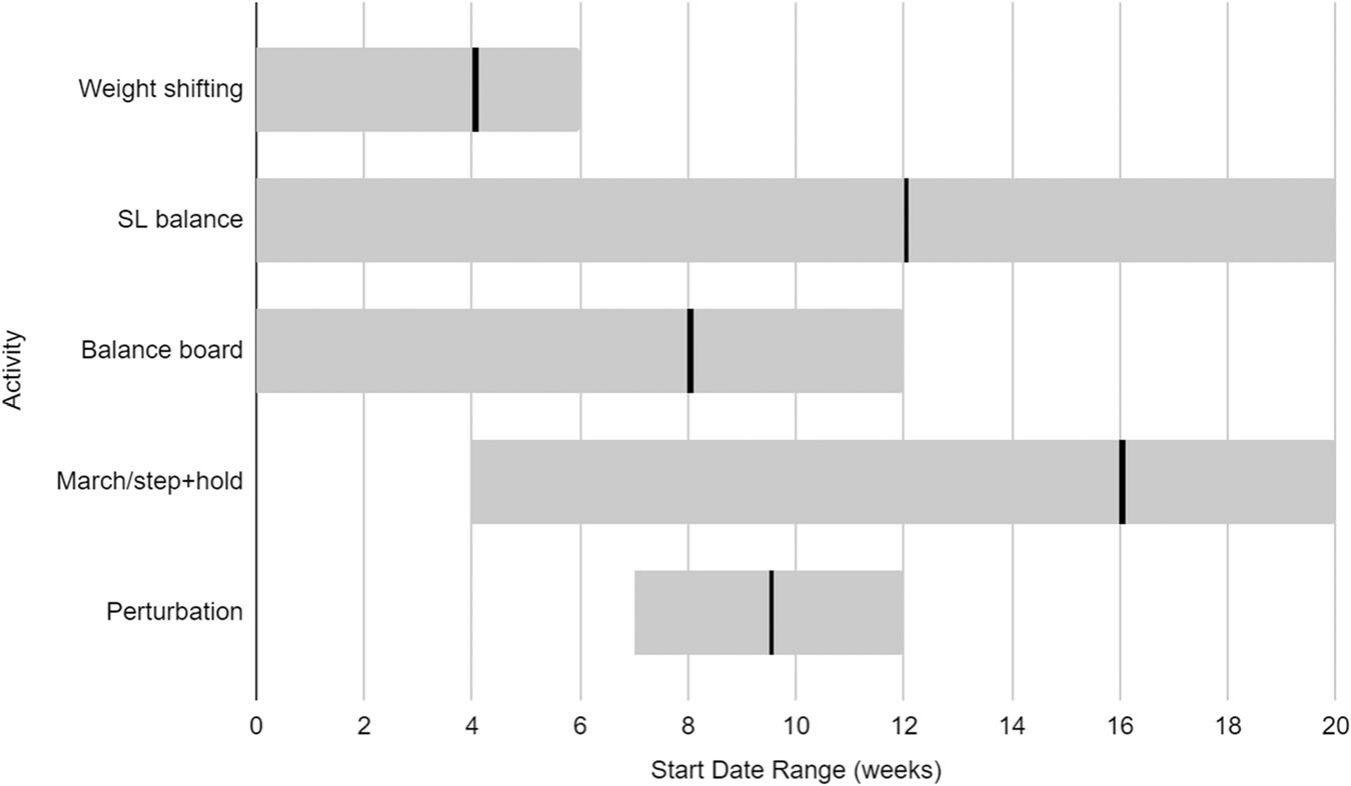
Proprioception and balance exercise timeline. Black lines indicate medians, with ranges shown in grey. SL, single leg.

Timing recommendations varied: Five of nine protocols (71.4%) suggested starting general balance training at 4–8 weeks, while others recommended between 1–4 weeks (14.3%) or 6–12 weeks (14.3%). Proprioceptive training start times also varied: Three (33.3%) suggested 1–6 weeks, four (44.4%) suggested 8–16 weeks and two (22.2%) suggested 13–20 weeks (Figure 7).

### Functional testing

**Figure 8:**
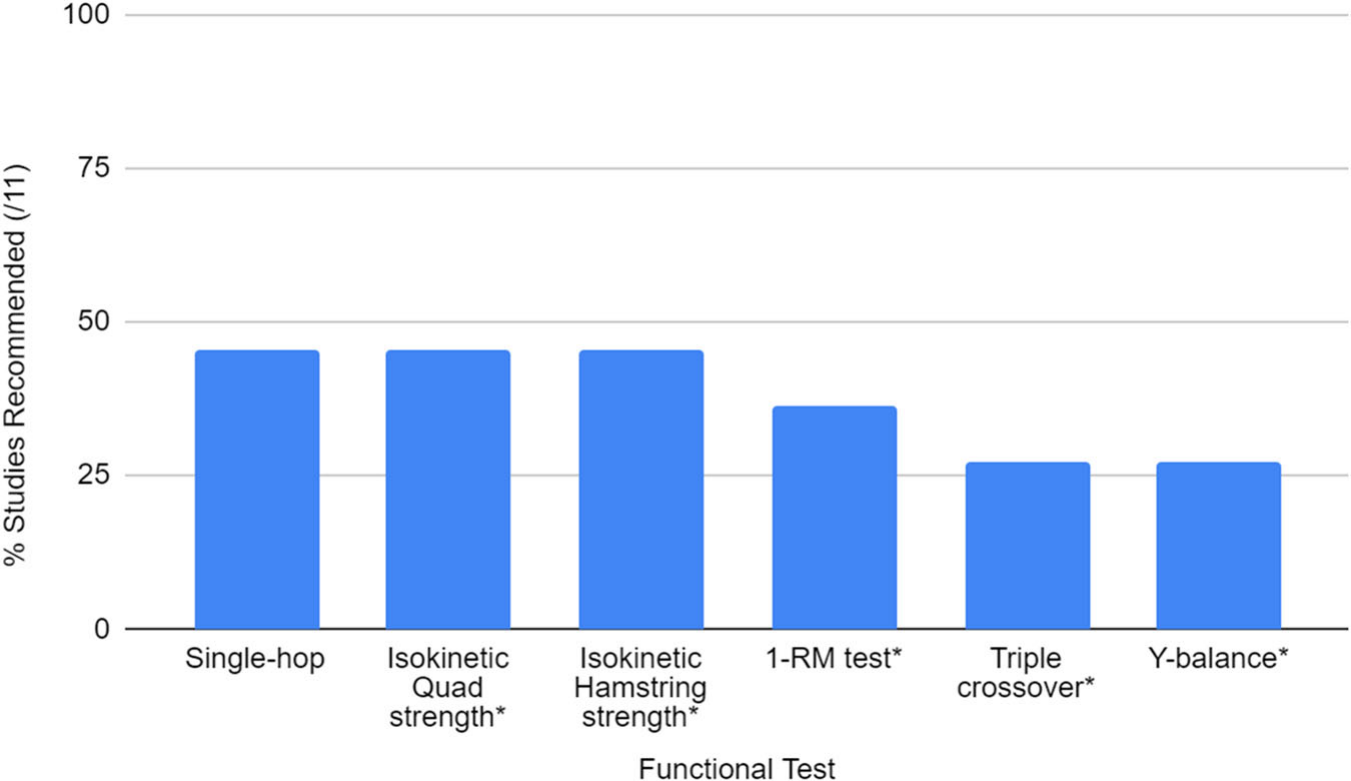
Functional testing recommendation. *Several of the studies in these categories did not mention the timing of initiation of the functional tests. RM, repetition maximum.

**Figure 9:**
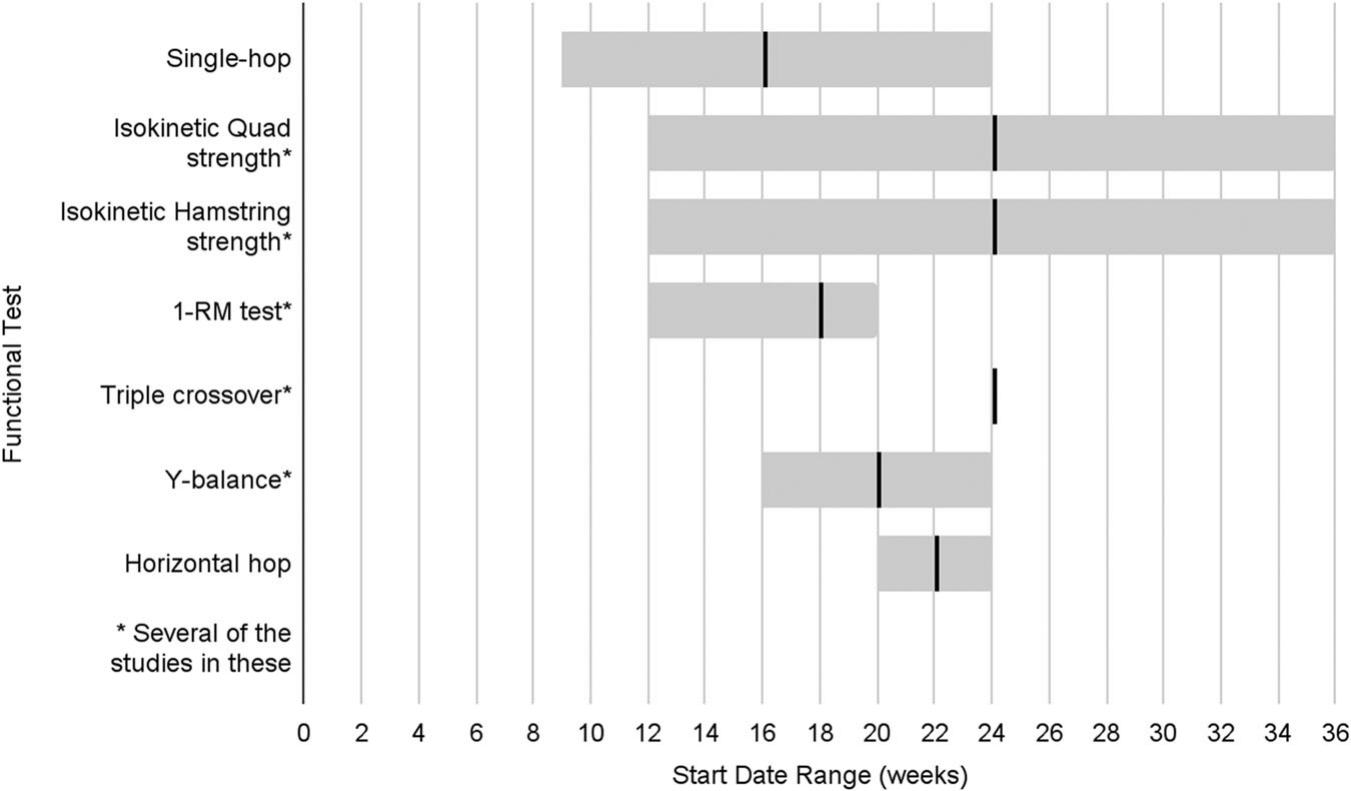
Functional testing timeline. Black lines indicate medians, with ranges shown in grey. *Several of the studies in these categories did not mention the timing of initiation of the functional tests. RM, repetition maximum.

Eleven protocols (68.8%) recommended at least one functional test postoperatively (Table 6; Figure 8). Nine functional tests were identified, with the single‐hop test (45.5%), quadriceps isokinetic strength (45.5%) and hamstring isokinetic strength (45.5%) being the most common. Ten (90.9%) of the eleven protocols recommended some variation of hop testing, with start times ranging from 9 weeks to 6 months. Isokinetic strength testing was recommended by 7 of the 11 protocols (63.6%), specifically for quadriceps (57.1%) and hamstrings (42.8%). Four protocols recommended single repetition maximal strength testing, with start times varying from 12 to 20 weeks (Figure 9). Two protocols suggested using the Biodex machine for progression assessment.

**Table 6:** Functional testing recommendations.

Functional test	% Studies recommended (/11)	Start date range (weeks)	Individual ranges	Median (weeks)
Single‐hop	45.5	9–24	[9–12]; [10]; [16]; [16–22]; [24]	16
Isokinetic quad strength^a^	45.5	12–36	[12]; [13–26]; [24]; [24–36]	24
Isokinetic hamstring strength^a^	45.5	12–36	[12]; [24]; [13–26]; [24–36]	24
1‐RM test^a^	36.4	12–20	[12]; [16–20]; [16–20]	18
Triple crossover^a^	27.3	24	[24]	NA
Y‐balance^a^	27.3	16–26	[16–20]; [20–26]	20
Horizontal hop	18.2	20–24	[20]; [24]	22

Abbreviation: NA, not applicable; RM, repetition maximum.

a Several of the studies in these categories did not mention the timing of initiation of the functional tests.

### RTS/play

**Figure 10:**
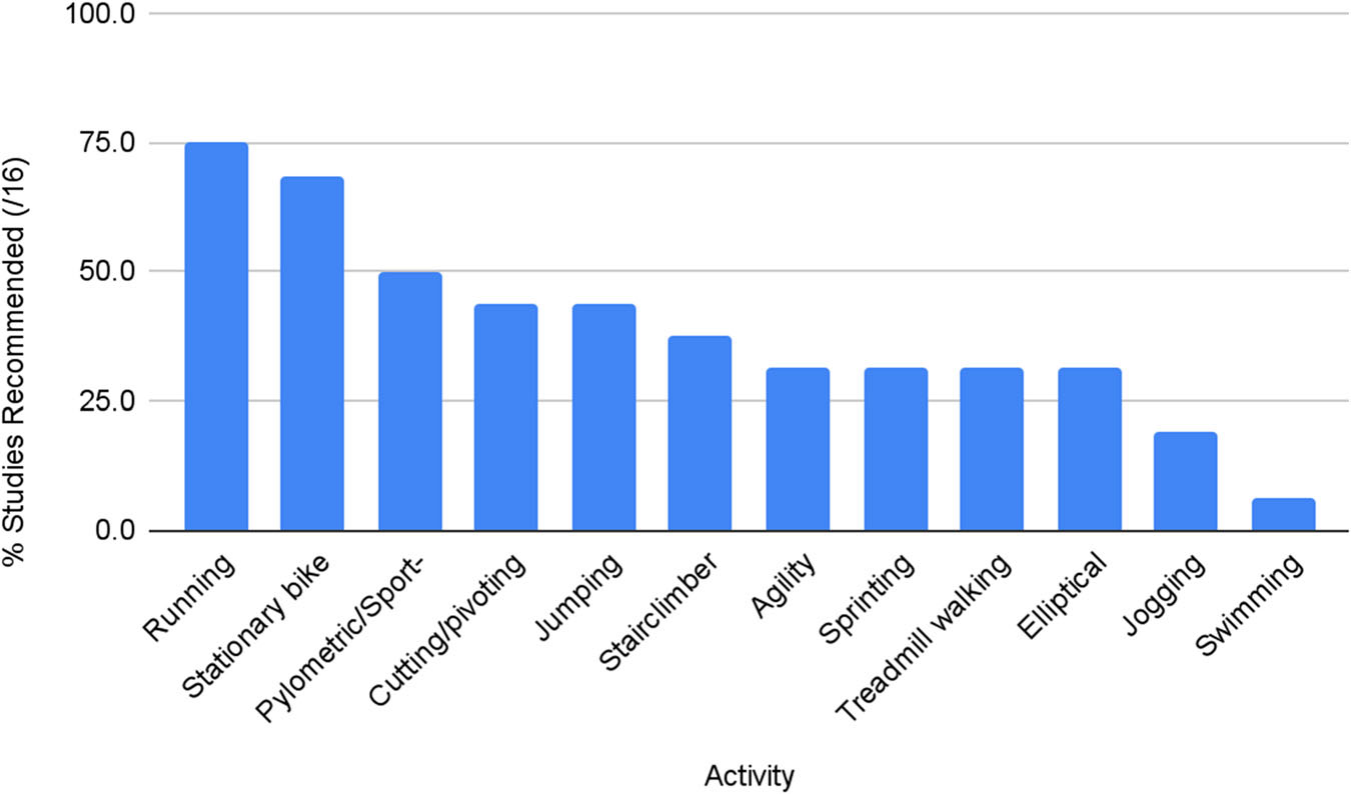
Return to sport/play recommendations.

**Figure 11:**
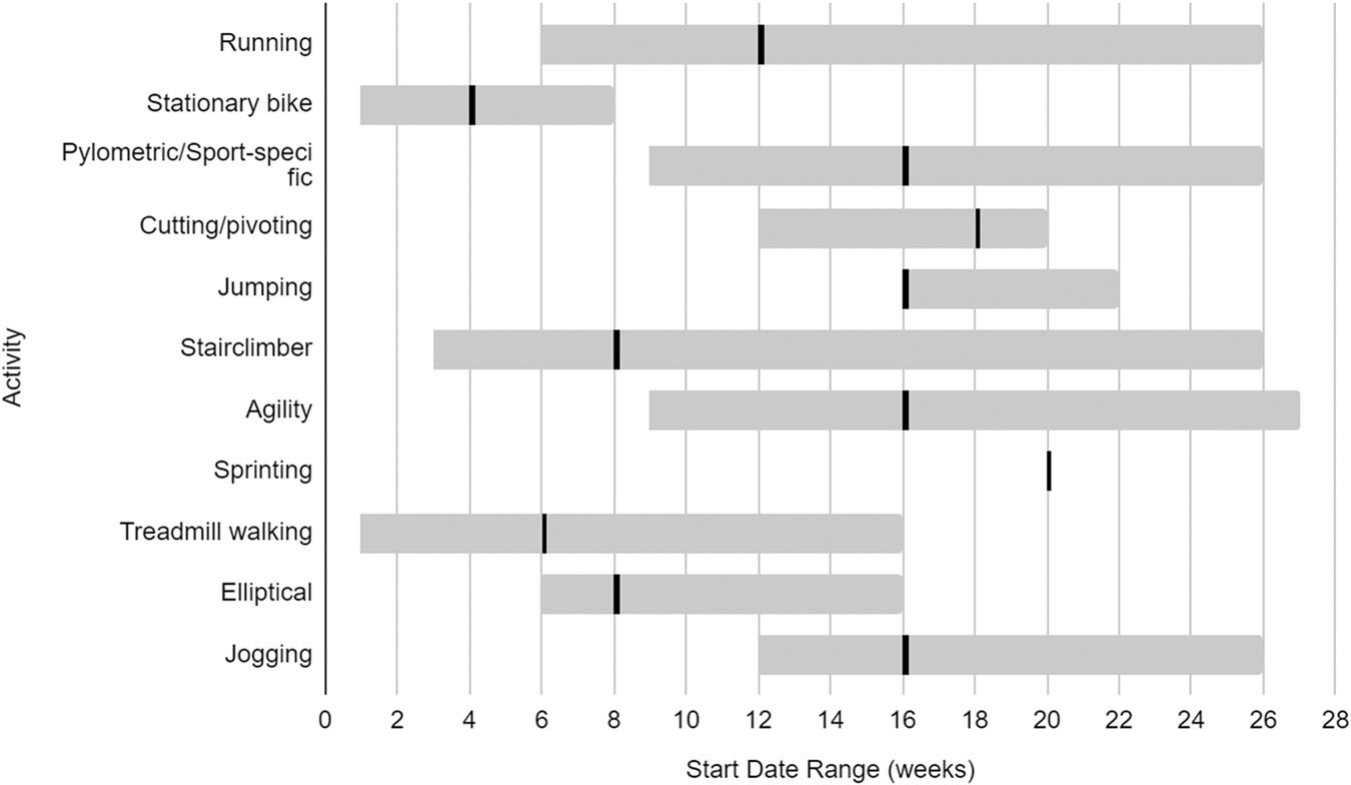
Return to sport/play timeline. Black lines indicate medians, with ranges shown in grey.

All 16 protocols included time‐ and criteria‐based guidelines for returning to sport (RTS) or activity (Table 7; Figure 10). Time‐based recommendations for the earliest consideration of RTS were variable: Seven protocols (43.8%) recommended after 6 months, four protocols (25.0%) after 5 months and three protocols (18.8%) after 9 months. Two protocols (12.5%) began their RTS phase much earlier, 3 months post‐operation (Figure 11).

**Table 7:** Return to sport/play recommendations.

Activity	% Studies recommended (/16)	Start date range (weeks)	Individual ranges	Median (weeks)
Running	75.0	6–26	[6–8]; [8–12]; [12]; [12]; [12–16]; [12–16]; [12–16]; [12–18]; [16]; [16–20]; [16–20]; [13–26]	12
Stationary bike	68.6	1–8	[1]; [2–4]; [2–6]; [2–6]; [3–6]; [4–6]; [4–6]; [4–8]; [4–8]; [4–8]; [4–8]	4
Pylometric/sport‐specific	50.0	9–26	[9–12]; [12–16]; [12–18]; [16–20]; [16–20];[20]; [20]; [13–26]	16
Cutting/pivoting	43.8	12–20	[12–18]; [12–18];[16–20]; [16–20]; [20]; [20]; [20]	18
Jumping	43.8	16–22	[16], [16]; [16]; [16]; [16–20]; [16–22]; [22]	16
Stairclimber	37.5	3–26	[3–5]; [6–12] [8]; [8]; [12–16]; [12–16]	8
Agility	31.3	9–27	[9–12]; [12–18]; [16]; [16–20]; [13–26]	16
Sprinting	31.3	20	[20]; [20] [20]; [20]; [20]	20
Treadmill walking	31.3	1–16	[1–4]; [6]; [6–8]; [6–12]; [4–16]	6
Elliptical	31.3	6–16	[6]; [7–12]; [8]; [12]; [12–16]	8
Jogging	18.8	12–26	[12–16]; [16]; [13–26]	16
Swimming	6.3	NR	NA	NA

Abbreviations: NA, not applicable; NR, not reported.

There was considerable variability among the 16 protocols, including criteria‐based RTS recommendations. Both functional strength testing (50.0%) and hop testing (50.0%) were commonly included amongst the 16 protocols, although specific tests and criteria required for RTS varied. More general recommendations, such as having patients undergo a Functional Sporting Assessment (FSA)(43.8%) or clearance from the operating surgeon (43.8%), were also common.

All 16 protocols included activities to aid rehabilitation and RTS, with running (75%), stationary bikes (68.6%) and plyometric/sport‐specific drills (50%) being the most common. Early recovery activities included stationary bike (1–8 weeks), treadmill walking (1–16 weeks) and stair climbing (3–26 weeks). Later recovery activities included running (6–26 weeks), elliptical (6–12 weeks), plyometrics (9–26 weeks) and agility (9–27 weeks). More complex activities such as cutting/pivoting (12–20 weeks), jumping (16–22 weeks) and sprinting (20+ weeks) were recommended later in the postoperative period, often with contingencies or prerequisite testing.

## DISCUSSION

Across the publicly available postoperative rehabilitation protocols for isolated QT‐ACLR, several consistent trends emerged: use of a hinged brace locked in extension for at least 2–4 weeks; immediate initiation of icing, cryotherapy and patellar mobilizations; NMES, if used, within the first 4 weeks; achievement of extension ROM by 2–4 weeks and flexion ROM by 3–4 months; strengthening, balance and proprioceptive training beginning 1–3 months postoperatively; and return‐to‐play testing at 5–9 months using either time‐ or criteria‐based approaches. Despite these commonalities, there was wide variability in the timing, detail and progression of rehabilitation activities. This lack of consensus may hinder patient outcomes and underscores the need for standardized, evidence‐based protocols specific to QT‐ACLR.

### Comparison with HT and PT protocols

Many of the identified QT protocols mirrored those historically developed for PT and HT autografts, suggesting that rehabilitation principles are often transferred rather than tailored. Weight‐bearing and ROM milestones were similar across graft types, aligning with evidence that accelerated rehabilitation is safe following PT and HT ACLR [6, 8, 10, 15, 23].

However, QT autografts involve unique considerations such as extensor mechanism harvest, quadriceps weakness and possible anterior knee pain. These features may warrant graft‐specific rehabilitation adjustments. Recent literature has underscored the importance of closely monitoring quadriceps recovery, particularly when a bone plug is harvested [21]. Other reviews have highlighted the paucity of standardized QT‐specific rehabilitation protocols, despite overall outcomes being comparable to PT and HT autografts [26].

### Effect of graft configuration on rehabilitation

None of the protocols stratified rehabilitation recommendations by QT configuration (full vs. partial thickness) or graft type (bone‐block vs. all–soft‐tissue). This consistency aligns with prior evidence showing that these technical variations (full vs. partial thickness [12] or bone‐block vs. all–soft‐tissue [5]) do not significantly affect biomechanical integrity or clinical outcomes following QT‐ACLR. These findings suggest that postoperative rehabilitation principles can remain largely uniform regardless of the specific QT harvest technique.

### Prehabilitation

Only three protocols addressed prehabilitation. Given the risk of quadriceps inhibition after QT harvest, prehabilitation may be particularly valuable for this graft. A recent systematic review concluded that prehabilitation before ACLR is safe and provides both short‐ and long‐term benefits [25]. This supports incorporation of structured prehabilitation into QT‐ACLR rehabilitation pathways.

### Adjunctive therapies, bracing and cost‐effectiveness

Although several reviews show no consistent benefit from routine bracing after ACL reconstruction, most available data are from mixed graft types [1, 24]. In QT‐ACLR, unique factors such as early quadriceps inhibition and slower strength recovery may justify short‐term protective bracing to support extension and gait stability [11]. From a cost perspective, brief use of a hinged brace, especially if covered by insurance, likely carries minimal financial burden compared with the potential consequences of early graft failure or extensor lag. While large studies still question routine bracing, these graft‐specific considerations make a cautious and time‐limited approach reasonable until stronger evidence emerges.

Most protocols recommended other adjunctive therapies, such as NMES or cryotherapy, but without clear evidence to guide their optimal use. Variability in adjunctive therapy recommendations may also affect patient adherence. Protocols requiring expensive devices or multiple daily interventions may reduce compliance, while simpler approaches may improve engagement.

### Open‐ versus closed‐chain strengthening

Only 2 of the 15 protocols explicitly mentioned open‐chain exercises, whereas seven protocols mentioned closed‐chain exercises. Closed‐chain movements (e.g., leg press, step‐ups) were introduced earlier, typically within 2–6 weeks (median 6), whereas open‐chain knee extensions appeared later, around 4–12 weeks (median 8). This sequencing aligns with current rehabilitation principles favouring early closed‐chain loading to minimize anterior shear forces, followed by cautious, limited‐arc open‐chain work once full extension and good quadriceps control are regained [15]. For QT grafts, where extensor mechanism weakness is common [11], delaying resisted open‐chain exercises until 6–8 weeks postoperatively and beginning with low‐load arcs from 90° to 45° may help protect the graft while restoring strength.

### BFR

Only one protocol recommended BFR but did not include specific BFR parameters or guidance. Although recent studies suggest potential benefits for early quadriceps strength and muscle preservation after ACL reconstruction [17], evidence specific to QT autografts remains limited and inconsistent [9].

### Telehealth and rehabilitation delivery

The rise of telehealth ACL rehabilitation provides both challenges and opportunities [7, 22]. Most current QT protocols are not designed with virtual delivery in mind. Standardized, simplified and outcome‐focused protocols could enhance feasibility of remote rehabilitation, potentially improving access while maintaining adherence.

### Psychological and mental health factors

None of the reviewed protocols addressed mental health or psychological readiness, despite growing recognition of these factors in ACLR recovery. Fear of reinjury, anxiety and kinesiophobia significantly influence outcomes, as shown in a recent systematic review [16]. Integrating psychological assessment and support into QT rehabilitation could improve RTS success.

### Surgeon‐specific variability

Heterogeneity in QT protocols may also reflect surgeon preference rather than evidence‐based consensus. Surgeons' training, experience and perceptions of risk likely drive differences in rehabilitation timelines and adjunctive therapy use. This underscores the need for guideline development to reduce variability driven by personal practice.

### Limitations

This study has several limitations. First, it is acknowledged that by intentionally excluding non‐ERAS/CaRMS‐accredited sources, this study may omit community‐driven protocols. Second, the absence of outcome data linked to protocols prevents assessment of their efficacy. Third, while focusing on isolated QT‐ACLR increases methodologic purity, most ACLR cases involve concomitant injuries such as meniscal tears, which often necessitate altered rehabilitation (e.g., delayed weight bearing or restricted flexion). Fourth, only skeletally mature patients were represented. With QT increasingly used in adolescents, unique challenges such as compliance and adherence must be considered. Studies such as the SQuASH (soft‐tissue quadriceps autograft ACL‐reconstruction in the skeletally immature versus hamstrings) trial highlight the need for age‐appropriate QT rehabilitation strategies [2].

## CONCLUSION

While QT‐ACLR rehabilitation protocols share some key themes such as early weight bearing, immediate cryotherapy and patellar mobilization, NMES in the early postoperative period and RTS testing around 5–9 months, substantial variability persists in timing, exercise progression and adjunctive therapy use. This variability may reduce patient adherence, increase costs and reflect surgeon preference more than evidence‐based practice. Compared with PT and HT protocols, QT rehabilitation often appears extrapolated rather than specifically adapted, despite graft‐specific risks to the quadriceps and extensor mechanism. Future work should prioritize the development of graft‐specific, evidence‐based QT‐ACLR protocols; evaluate prehabilitation and psychological readiness; assess cost‐effectiveness of adjunctive therapies and consider telehealth as a delivery platform.

## AUTHOR CONTRIBUTIONS

David Slawaska‐Eng conceived and designed the study, performed the statistical analysis and drafted the manuscript. Emily Zhang and Kanika Tibriwal assisted with data collection. Caitlin Svendsen, Dan Cohen and Lauren Gyemi contributed to data analysis and manuscript writing. Sachin Tapasvi, Matthieu Ollivier and Darren de SA contributed to manuscript editing and critical revisions. All authors reviewed and approved the final manuscript.

## CONFLICT OF INTEREST STATEMENT

Sachin Tapasvi is a consultant for Microport Orthopaedics, Newclip Technic and Smith & Nephew; receives royalties from Jaypee Publications; serves on the Board of *ISAKOS*; and is Associate Editor of *JISAKOS*. Matthieu Ollivier receives royalties from Stryker and Newclip Technic; is a consultant for Arthrex, Stryker and Newclip Technic; and serves as a Section Editor for Knee Surgery, Sports Traumatology, Arthroscopy (KSSTA). Darren de SA is a consultant for Pendopharm; serves as Associate Editor for KSSTA; and has received multiple peer‐reviewed research grants from the Canadian Institutes of Health Research (CIHR), Arthroscopy Association of North America (AANA), Physicians Services Incorporated Foundation, McMaster Surgical Associates and other organizations totalling over CAD $1.2 million. The remaining authors declare no conflict of interest.

## ETHICS STATEMENT

Ethics approval was not required for this study as all data were obtained from publicly available sources and did not involve human participants directly or identifiable private information. Patient consent was not required as only publicly available information was used.

## Data Availability

The data analysed in this study are publicly available and can be accessed from publicly accessible sources. No new data were generated during this study. Analysed raw data from the current study are available from the corresponding author on reasonable request.
